# Isoflavone Supplements for Menopausal Women: A Systematic Review

**DOI:** 10.3390/nu11112649

**Published:** 2019-11-04

**Authors:** Li-Ru Chen, Nai-Yu Ko, Kuo-Hu Chen

**Affiliations:** 1 Department of Physical Medicine and Rehabilitation, Mackay Memorial Hospital, Taipei 10449, Taiwan; gracealex168@gmail.com (L.-R.C.); Naiyuko@gmail.com (N.-Y.K.); 2 Department of Mechanical Engineering, National Chiao-Tung University, Hsinchu 300, Taiwan; 3 Department of Obstetrics and Gynecology, Taipei Tzu-Chi Hospital, The Buddhist Tzu-Chi Medical Foundation, Taipei 23142, Taiwan; 4 School of Medicine, Tzu-Chi University, Hualien 970, Taiwan

**Keywords:** isoflavone, daidzein, genistein, equol, menopause

## Abstract

Isoflavones have gained popularity as an alternative treatment for menopausal symptoms for people who cannot or are unwilling to take hormone replacement therapy. However, there is still no consensus on the effects of isoflavones despite over two decades of vigorous research. This systematic review aims to summarize the current literature on isoflavone supplements, focusing on the active ingredients daidzein, genistein, and S-equol, and provide a framework to guide future research. We performed a literature search in Ovid Medline using the search terms “isoflavone” and “menopause”, which yielded 95 abstracts and 68 full-text articles. We found that isoflavones reduce hot flashes even accounting for placebo effect, attenuate lumbar spine bone mineral density (BMD) loss, show beneficial effects on systolic blood pressure during early menopause, and improve glycemic control in vitro. There are currently no conclusive benefits of isoflavones on urogenital symptoms and cognition. Due to the lack of standardized research protocols including isoflavone component and dosage, outcomes, and trial duration, it is difficult to reach a conclusion at this point in time. Despite these limitations, the evidence thus far favors the use of isoflavones due to their safety profile and benefit to overall health.

## 1. Introduction

Menopause is a biological process that can cause various troublesome symptoms such as hot flashes and emotional changes, but can also increase mortality risk due to subsequent osteoporosis and reduced metabolism. Hormone replacement therapy (HRT) would be the most intuitive way to combat these changes; however, the 2002 Women’s Health Initiative (WHI) study showed that hormone replacement therapy increased the risk of breast cancer, stroke, and coronary heart disease in healthy postmenopausal women [1]. Since then, healthcare professionals and women have been seeking alternative therapies. In Canada for example, it has been found that 60% to 90% of women would consider taking complementary and/or alternative medicine (CAM) for menopausal symptoms, but are concerned about the efficacy and cost [2]. Many patients taking CAM choose not to tell their doctors because they feel doctors are not knowledgeable enough or are biased against CAM [3].

Vasomotor symptoms (VMS) such as hot flushes and sweating, are very common in menopausal populations and can cause physical and mental discomfort [4]. Estrogen remains the most effective therapy for hot flashes and is approved by the U.S. Food and Drug Administration (FDA) [5]. Although some women may prefer lifestyle modification, there is no evidence that lowering the room temperature, exercising, or avoiding triggers such as alcohol and spicy foods can improve hot flashes [5]. Currently, HRT is indicated for the prevention of osteoporosis and relief of the VMS and vulvovaginal atrophy associated with menopause. Moreover, HRT increases bone mineral density (BMD) and reduces the incidence of osteoporotic fractures [6]. Due to the serious side effects mentioned above, HRT should be given in the lowest effective doses for the shortest duration to meet the treatment goals. In fact, the FDA recommends that approved non-estrogen treatments can first be carefully considered before relying solely on HRT for osteoporosis prevention [7]. VMS seriously affects quality of life during menopause and the role of HRT is still fundamental. For affected women who cannot use HRT, evidence has shown that acupuncture, hypnosis, paced respiration, cognitive behavioral therapy, combined preparations of black cohosh, and omega-3 supplements may significantly reduce vasomotor symptoms [4].

An alternative to traditional HRT is tibolone (Livial^®^, 2.5 mg tablet), a new regimen consisting of a synthetic steroid hormone broken down by the body’s metabolism into three compounds that act similarly to naturally-produced estrogen, progesterone, and testosterone [6]. These metabolites are tissue-specific, having estrogenic effects on bone, vaginal, and breast tissues, progesterone effects on the endometrium, and androgenic effects (like testosterone) on the brain and liver. In this way, tibolone helps restore the hormonal balance to relieve menopausal symptoms, such as vaginal atrophy, loss of bone density, and osteoporosis, as well as other symptoms, such as hot flashes and decreased libido. Tibolone also lowers cholesterol levels, which tend to increase after menopause [6].

Selective Estrogen Receptor Modulators (SERMs), such as Raloxifene (Evista^®^), can be used for both the prevention and treatment of osteoporosis in postmenopausal women. SERMs are non-steroidal compounds with tissue-specific actions, which induce a different response in the ER from estradiol and enhance osteoclast apoptosis [6]. In postmenopausal women, SERMs reduce the risk of vertebral fractures by approximately 30% in patients with a prior vertebral fracture and approximately 55% in patients without a prior vertebral fracture over three years, but do not protect against nonvertebral or hip fractures [7]. Using SERMs do not reduce the risk of coronary heart disease; moreover, they increase hot flashes and also the risk of deep vein thrombosis to a degree similar to that observed with estrogen [7].

In recent years, nutraceuticals such as phytoestrogens and herbal derivatives have gained popularity due to their claimed ability to relieve menopausal symptoms. Nutraceuticals are defined as foods, parts of foods, or botanicals that provide medical and health benefits, such as the prevention and treatment of disease [8]. Herbal remedies are frequently used to alleviate menopausal symptoms and may be effective for treating acute menopausal syndrome through different mechanisms [8]. Among them, *Actaea racemosa* can be used for the treatment of menopause symptoms such as hot flash, insomnia, irritability, but also musculoskeletal pain; *Ginkgo biloba* is effective on attention disorders in postmenopausal women; *Panax ginseng* alleviates sleep disorders, depression, and improves sexual function; *Valerian officinal* is useful for hot flashes, anxiety, sleep disorders, and dysmenorrhea [8]. Not similar to phytoestrogens, herbal derivatives including *Actea racemosa*, *Ginkgo biloba,* and *Valerian officinal* are acting through estrogen-independent pathways to alleviate menopausal symptoms. One of the major problems with herbal therapies is that people usually take supplement pills that are not prepared by trained herbalists. As herbal supplements are not strictly regulated like prescription drugs, the quality and safety may vary between brands or even between bundles of the same brand [8]. These compounds may also interact with prescription drugs, resulting in dangerous adverse events [8].

Epidemiological studies have suggested that there is a link between countries that consume soy and decreased VMS, namely hot flashes [9]. Soy intake is estimated to be four to nine times greater in Asian countries such as Japan, Korea, China, Taiwan, and Indonesia, than in Western countries such as the United States, and women in Asian countries report a much lower incidence of hot flashes (10–25%) compared to women in Western countries (60–90%) [10]. However, the calculation of incidences may be biased by the declaration frequency.

Isoflavones, compounds abundant in soybeans, are thought to be responsible for exerting estrogen-like effects, thereby relieving menopausal symptoms. There are two types of estrogen receptors: ERα, the predominant form in the breast and uterus, and ERβ, the predominant form in the cardiovascular system, urogenital tract, and bone [3]. Isoflavones bind weakly to ERα; the affinity of isofolavones to ERβ is higher. The estimated estrogenic effects of different isoflavones using human cell cultures in vitro have shown that the relative potencies are estradiol 100, genistein 0.084, equol 0.061, and daidzein 0.013, respectively [11]. However, isoflavones can circulate at 10,000 times the concentration of estradiol and achieve greater binding potential through abundance [12]. For example, in estrogen-deficient female aromatase knockout mice, isoflavones act as estrogens and improve ovarian morphology [13].

Many studies have been published on the effects of isoflavones; however, there is still no consensus despite over two decades of vigorous research. Systematic reviews and meta-analyses have pointed out that this is because trials vary in design, isoflavone formulation, dosage, and duration, and are limited by small sample size and high drop-out rates [14]. Furthermore, the age of the women is highly variable and the time since the onset of menopause is highly variable. Based on previous research which explored the nature of isoflavones and their mechanism of action, this systematic review aims to summarize the current literature on isoflavone supplements, focusing on the active ingredients daidzein, genistein, and S-equol, and provide a framework to guide future research. In order to review the literature, searches for possible publications were performed using the database of Ovid Medline from its inception to August 2019. The search strategy for the database was personalized and included a combination of medical subject heading (MeSH) terms and entry terms to meet the definitions of isoflavone and menopause. Only original articles or systemic reviews which were published in English in peer-reviewed journals were considered for further inclusion. Using the keywords “isoflavone”, “menopause”, and their combination, we performed a literature search in Ovid Medline, which yielded 95 abstracts and 68 full-text articles for further analyses and interpretation.

## 2. Terminology

### 2.1. Isoflavones (Genistin, Daidzin), Genistein, Daidzein

**Figure 1:**
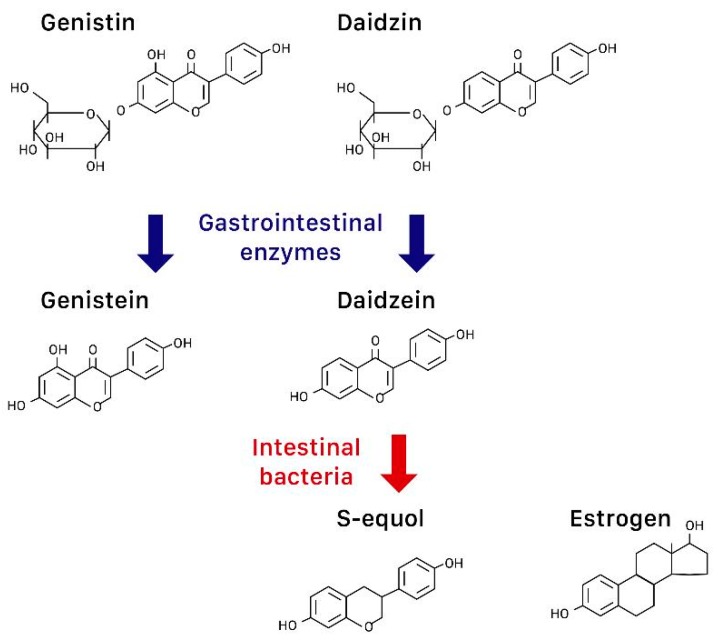
The chemical structures of genistein and its precursor genistin, daidzein and its precursor daidzin, s-equol, and estrogen.

The terms phytoestrogens (or plant estrogens), isoflavones, and soy are often used interchangeably in the literature, but they are not the same. More accurately, phytoestrogens are plant compounds that have estrogen-like effects and isoflavones such as genistin and daidzin are a type of bioflavonoids that are found in both plants and animals [15]. Isoflavones are abundant in foods including soybeans, red clover, and alfalfa. After isoflavone metabolization in the human gut, the precursors genistin and daidzin become the “aglycones” genistein and daidzein, respectively, through the effect of gastrointestinal enzymes [16] (Figure 1). Fermentation of soybeans (e.g., to make miso) concentrates isoflavones, while processing methods that remove fat, taste, and color tend to remove isoflavones [17].

### 2.2. Equol

In over 50% of Asians but less than 20% of Caucasians, their intestinal bacteria can further convert daidzein to S-equol, a compound structurally similar to estrogen [18] (Figure 1). S-equol also preferentially binds to ERβ, but has a higher transcriptional expression than isoflavones [17].

Research has found that for non-equol producers, even if they consume adequate daidzein, the efficacy of alleviating menopausal symptoms may still be limited [18]. In an observational study of 364 women, only equol-producers benefited from dietary daidzein in reducing vasomotor symptoms [19]. Ingesting probiotics has failed to stimulate S-equol production in some studies [20], while a recent randomized controlled trial (RCT) showed red clover extract with probiotics effectively reduced VMS [12]. From fecal cultures of equol-producing women, equol-producing bacteria such as *Lactococcus garvieae* has been isolated [20,21], and a Japanese RCT showed that equol supplementation in non-equol producers successfully alleviated mood-related symptoms [22].

## 3. Effects of Isoflavones on Menopausal Syndromes and Others

### 3.1. Hot Flashes

VMS (hot flashes and night sweats) are perhaps the most immediate and troublesome consequence of menopause, and one of the main reasons menopausal women seek medical help. However, as the frequency and severity of hot flashes are subjective and symptoms often resolve over time without treatment [16], VMS are difficult to quantify. A placebo effect is unavoidable, as illustrated in the 24-week study done by St. Germain et al. in 2001, which showed hot flash decline in all patients, whether they received isoflavone-rich soy, isoflavone-poor soy, or whey protein [23]. Tice et al. also found no difference in the frequency of hot flash after 12 weeks of treatment with either isoflavones or placebo [24].

Nonetheless, more recent research favors the use of isoflavones for treating hot flashes. Cancellieri et al. found that using an herbal supplement containing 72 mg of isoflavones from soy beans and red clover for 6 months significantly reduced hot flashes using the Kupperman Menopause Index [25]. Another small-scale prospective study of 51 healthy postmenopausal women also found a 57% reduction in the frequency and severity of hot flashes (on a five-point self-rating scale) after taking 60 mg of isoflavones daily for 12 weeks [26].

To cater to the Western diet, Welty et al. gave subjects soy nuts as a substitution for non-soy protein for 8 weeks, and found over 40% reduction in hot flashes [9]. This was regardless of equol-producing status [9], suggesting a palatable way for Western women to achieve the same isoflavone benefits as Asian women through diet. However, synthetic isoflavones may still be more effective than soy isoflavones in reducing hot flashes, as demonstrated in a systematic review [27].

The optimal dosage of isoflavones has also been an area of interest. In 1999, Washburn et al. found splitting the dose of soy supplement to twice-daily decreased the severity of hot flashes more than giving the total amount in one dose, suggesting consistent circulating levels of phytoestrogens to be more effective [28]. This finding can also be inferred from another study where once-daily dosing of either 40 mg or 60 mg of daidzein-rich isoflavone aglycones reduced the frequency of hot flash by the same amount [29].

The combined effect of soy isoflavones and inulin (SII) has also been studied. In an observational prospective, multicentric study, Cianci et al. evaluated the effect of soy isoflavones and inulin (SII) on hot flashes and quality of life in peri/postmenopausal women treated or untreated with a product consisting of a mixture of calcium (500 mg), vitamin D3 (300 IU), inulin (3 g) and soy isoflavones (40 mg) [30]. They found the mean number of hot flushes declined by 2.8 (SD 3.7) in the intervention (SII) group and 0.0 in the untreated one. The corresponding values after six months were −3.7 (SD 2.7) in the intervention (SII) group and −0.9 (SD 5.3) in the control group (*p* = 0.02). This observational trial suggests there may be beneficial effects of combining 40 mg of isoflavone and inulin given as a daily dietary soy supplement in the management of menopausal symptoms [30].

However, when compared to HRT, isoflavones still fall short. In an RCT comparing 45 mg of isoflavones twice daily, low-dose HRT (1 mg of estradiol and 0.5 mg of norethisterone acetate), and placebo using the Menopause Rating Scale, both treatments were superior to placebo, but HRT was superior to isoflavones [31]. A meta-analysis of 51 RCTs also found a statistically significant difference between the effects of HRT and soy extracts on hot flashes using indirect comparison [32].

On the other hand, a 24-month study found that isoflavone tablets (containing 22.01 mg of daidzein, 13.54 mg of glycitein, and 4.96 mg of genistein) thrice-daily did not affect Menopause-Specific Quality of Life (MENQOL) measures significantly [33]. The results of the aforementioned studies reveal that isoflavones cannot totally replace traditional HRT in alleviating menopausal syndromes.

Studies have also pointed out that the ability of women to produce equol may be the major determinant of whether or not isoflavones can effectively reduce VMS. A systematic review and meta-analysis of RCTs assessed the efficacy of soy isoflavones and equol for alleviating menopausal symptoms (especially vasomotor symptoms) in postmenopausal women who were either equol producers or nonproducers [34]. The result of this meta-analysis revealed a significant benefit of equol for decreasing hot flash scores. This study concluded that supplementing equol to equol nonproducers significantly lowered the incidence and/or severity of hot flashes in menopausal women [34]. However, equol may not be the only factor because that the duration of menopause is highly variable, and that the estradiol receptors decrease with time from menopause.

In order to minimize subjectivity of the frequency and severity of hot flashes, skin conductance measurement has been used in some studies. Skin conductance quantifies the preceding small increase in core body temperature and subsequent sweat response [12]. Although Newton et al. found consistent results whether using a diary or a sternal skin conductance monitor [19], Lambert et al. found differences between subjective reporting of hot flashes using the Green Climacteric Scale and measuring hot flashes using 24 h ambulatory skin conductance [12]. In their study, red clover extract and probiotics significantly reduced hot flashes using the measurement of skin conductance but not Green Climacteric Scale, highlighting the need for an objective measure [12].

### 3.2. Bone Mineral Density

Another significant change that occurs during menopause is the loss of bone mineral density (BMD), causing osteoporosis. As there are high levels of ERβ in bone [15], isoflavones could theoretically prevent bone loss. Previous research has focused on ipriflavone, a synthetic isoflavone, but results have been inconclusive [16].

The spine in particular is thought to be the most sensitive to isoflavones because it has a higher content of trabecular bone compared to cortical bone. Trabecular bone has a higher expression of ERβ and a larger surface area for receptor binding [35]. The hip, on the other hand, contains a higher percentage of cortical bone, and is remodeled slower than the spine [36]. A meta-analysis showed significant attenuation of spinal bone loss after 6 months of over 90 mg/day of isoflavone supplement [36]. Amato et al. found that while 120 mg of isoflavones per day did not slow bone loss at regional bone sites, there was slowing of BMD loss [33]. A systematic review and meta-analysis published in 2017 reiterated that isoflavones attenuated BMD loss, but more so at the lumbar spine compared to the femoral neck, and isoflavones in aglycone form were more efficacious [35].

As bone remodeling takes around four to eight months, studies of longer duration could help elucidate more conclusive results [35]. In the meantime, even slight improvements in BMD may be beneficial for postmenopausal women who are not on hormone replacement therapy [16].

### 3.3. Cardiovascular Profile

Although there are many independent cardiovascular risks including age, the incidence of cardiovascular events in women also increases after menopause due to estrogen deficiency [37]. The lack of estrogen leads to a rise in low-density lipoprotein (LDL) cholesterol, endothelial dysfunction, and reduced carotid arterial pulsatility [38]. Isoflavones may be able to reduce cardiovascular risk by acting as estrogen substitutes.

As soy products do not contain cholesterol, they are generally regarded as healthy food, leading the United States Food and Drug Administration (FDA) to issue a statement saying soy protein may reduce the risk of heart disease [16]. However, habitual intake of soy in Western countries is low. A Dutch prospective study of 16,165 women followed up for a median of 75 months found no correlation between habitual phytoestrogen intake (including lignans and isoflavones) of the Western diet and cardiovascular disease risk, possibly due to the low overall isoflavone intake [37].

The mechanism of isoflavones in preventing cardiovascular events, if any, remains to be elucidated. Previous small-scale research has suggested that in perimenopausal and menopausal women, soy isoflavones may improve systemic arterial compliance, although they had no effect on plasma lipids [38]. A 2007 study similarly showed no difference in lipoprotein lipids after 12 weeks of treatment with 60 mg of isoflavones daily [26]. Therefore, isoflavones may exert their cardioprotective effects in ways other than lowering lipids [39].

It has been suggested that genistein and daidzein cause arterial relaxation through the release of nitric oxide [39]. However, Wong et al. found that 80 mg of soy isoflavones per day for six weeks had no significant effect on blood pressure [40]. In fact, in vessels with pre-existing atherosclerotic changes, animal studies have shown detrimental effects of soy and HRT. In ovariectomized and diet-induced atherosclerotic monkeys, neither soy nor HRT reduced myocardial ischemia/reperfusion injury, and the combination of both actually increased post-ischemic myocardial change [41]. Animal studies must be interpreted with caution however, and further research is required to reach a conclusion before advising women against consuming soy products once they experience a coronary event [39].

A recent RCT revealed that soy isoflavones reduce systolic blood pressure in early menopause. In women within two years of menopause, Sathyapalan et al. found that treatment with soy isoflavone supplements for six months reduced systolic blood pressure, though there were no changes in diastolic blood pressure or lipid parameters (total cholesterol, LDL, HDL, and triglycerides) [42]. The reduction in systolic blood pressure translated into reduced risk of cardiovascular disease using the Framingham equation [42]. According to the above findings, one can infer that the safest and most effective treatment window for cardiovascular disease would be early menopause, before critical atherosclerotic change.

It is also possible that isoflavones only play a small role in preventing cardiovascular events, and that the main benefits seen are from soy itself. Soy, in contrast to animal protein, contains minimal cholesterol and saturated fats [16], making it a more healthy way to meet daily protein requirements. Therefore, whether it is due to soy containing copious amounts of polyunsaturated fats, fiber, and vitamins [43], or simply because it displaces dietary animal protein intake [17], soy products are currently recommended by both the FDA and AHA (American Heart Association) for the benefit of cardiovascular and overall health [16,43].

### 3.4. Metabolic Syndrome

The slowing of metabolism after menopause leads to obesity, an important risk factor of cardiovascular diseases [43]. There has been evidence to suggest that isoflavones improve glycemic control and promote weight loss [44].

In obese menopausal women, research on the relationship of soy isoflavone supplement and weight loss has been limited. In a study that showed isoflavone and exercise led to a reduction of fat mass, the high drop-out rate and questionable compliance to treatment made interpretation of results difficult [43].

In vitro studies are more optimistic. Genistein, daidzein, and equol show binding affinity to and activation of peroxisome proliferator-activated receptor (PPAR)γ, a drug target for type 2 diabetes and other components of metabolic syndrome [44]. Compared to rosiglitazone, an established anti-diabetic drug, the maximal PPARγ activity of isoflavones ranged from 23% to 32% [44]. As red clover extracts do not cause weight gain like rosiglitazone does in human studies [44], isoflavones could potentially be used to treat metabolic syndrome through glycemic control without the side effect of weight gain.

### 3.5. Cancer Risk

Because isoflavones bind to estrogen receptors, there has been concern of isoflavones inducing estrogen-sensitive malignancies, particularly in women who are at high risk or survivors of breast cancer [43]. However, in vitro studies have shown that breast cancer proliferation is dependent on increased ERα activity, and ERβ seems to suppress ERα-induced cancer cell proliferation [45]. Therefore, it is postulated that isoflavones, by binding to ERβ, is cancer-protective. In addition, phytoestrogens have been found to lengthen the menstrual cycle, a possible mechanism that potentially protects against hormone-dependent cancers [16]. However, such a mechanism has not been demonstrated yet.

In studies with healthy women, isoflavones were associated with a decreased risk of estrogen-sensitive cancer. A case-control study in Japan found that the risk of breast cancer was inversely related to soy consumption in premenopausal women, but found no link in postmenopausal women [46]. Although soy isoflavones induced some benign clinical and non-significant ultrasonographic changes of the breast in one 12-week study, none of these changes warranted intervention or follow-up for at least three months after the study [47]. On the other hand, soy isoflavones did not seem to stimulate endometrial proliferation during short-term treatment [17]. On the contrary, a study conducted by Shike et al. has explored the effects of soy supplementation on gene expression in breast cancer. In this RCT, the authors found that gene expression associated with soy intake and high plasma genistein defines a signature characterized by overexpression of FGFR2 and genes that drive cell cycle and proliferation pathways. Therefore, they concluded that in a subset of women soy could adversely affect gene expression in breast cancer [48].

The limited research on breast cancer patients taking soy isoflavones after surgery has also been promising. Kang et al. found that in postmenopausal women with estrogen- and progesterone-receptor positive breast cancers receiving anastrazole therapy after surgery, a higher intake of soy isoflavone was associated with a lower risk of recurrence [49].

Finally, isoflavones have also been suggested to protect against colon cancer. Risk factors for colon cancer include obesity, high levels of cholesterol, and type 2 diabetes, all of which isoflavones potentially treat. Therefore, stating that isoflavones prevent colon cancer would be a reasonable hypothesis. A study in Korea found that high intake of soy foods or isoflavones was associated with an overall reduction of risk in colorectal cancer, although high intake of fermented soy paste seemed to increase the risk of colorectal cancer in men, possibly due to the high salt content [50]. Further research is required to increase the validity of this hypothesis. Soy fibers may also play a role is this prevention. A future comparison of soy vs. isoflavone rich extracts should highlight whether or not this is the case.

### 3.6. Female Urogenital Tract

Estrogen has been used to treat vaginal dryness and incontinence with varying degrees of success. ERα and ERβ have been identified in urogenital tissues (including bladder, urethra, levator ani, and vaginal mucosa) variably [51]. These tissues also contain the GPER that has a great affinity for genistein and daidzein, and the mechanism of continence remains unknown. Isoflavone treatment has been trialed on menopausal symptoms affecting the urogenital tract.

Reed et al. found no differences in the vaginal cytology of women taking black cohosh or dietary soy after one year [52], though the isoflavone content of the supplements was not clearly stated. A 10-year longitudinal study found that neither high nor low dietary isoflavone intake prevented stress or urge incontinence [51], though they did not take into account equol-producer status.

Burton and Wells pointed out in their study investigating phytoestrogens and the female genital tract that although there is no concrete evidence to date to suggest phytoestrogens affect the human female genital tract, this is due to the paucity of research rather than the lack of correlation, and urge further investigation into this matter [53].

Regarding the effects of nutraceuticals on the sexual function of menopausal women, one study was identified. This prospective, randomized, placebo-controlled, parallel-group study was conducted to evaluate the effect of a mixture of isoflavones, calcium vitamin D, and inulin in menopausal women. Before and after treatment, both the patients in the treatment group (taking oral preparations of isoflavones (40 mg), calcium (500 mg) vitamin D (300 UI) plus inulin (3 g)) and the control group (taking placebo) were assessed for quality of life and sexual function using the Menopause-Specific Quality of Life Questionnaire (MENQOL) and Female Sexual Function Index (FSFI) [54]. A significant reduction in MENQOL vasomotor, physical, and sexual domain scores and a significant increase in all FSFI domain scores were observed in the treatment group after 12 months. This study suggests that a combination of isoflavones, calcium, vitamin D, and inulin may exert favorable effects on menopausal symptoms, sexual function, and quality of life [54].

### 3.7. Cognition

Sparse research has been done on the effects of isoflavones on cognitive function in menopausal women, possibly because the natural aging process is an inevitable confounding factor. The North American Menopause Society concluded in 2011 that soy may benefit cognitive function in women younger than 65, but not older [17].

A 6-year longitudinal study found that among women of different ethnicities with varying amounts of isoflavone intake undergoing menopause, Asian women with high intake of isoflavone had better processing speed during perimenopause and postmenopause, but worse verbal memory during early perimenopause and postmenopause [55]. The overall cognitive effects were small, casting doubt over the significance of these findings. A systematic review of twelve RCTs suggested isoflavones and soy may improve cognition in postmenopausal women, but pointed out most of the available studies had serious methodological flaws [56].

### 3.8. Side Effects

Isoflavones were generally well-tolerated in all of the studies we found. Side effects were mild and mostly gastrointestinal, including nausea, bloating, diarrhea, and constipation [1].

There has been concern over S-equol due to previous animal studies. Reproductive abnormalities were found in sheep ingesting red clover, and captive cheetahs fed soy-containing diets became infertile [20]. Ovariectomized Sprague-Dawley rats fed high-dose equol showed clear mammotropic effects after 3 months [57]. However, in humans, S-equol appears to be safe on the human uterus in all of the studies we identified for this review.

Table 1 is a brief summary showing current evidence of studies regarding the effects of isoflavones on menopausal syndromes and others.

## 4. Discussion

The common finding in all of the research included in this review is that past studies have shown high heterogeneity, making it difficult to draw conclusions [58,59,60]. In 2011, NAMS (the North American Menopause Society) suggested a trial-and-error approach to prescribing isoflavones for menopausal symptoms—initial treatment with high dose isoflavones (50 mg/day or higher) for 12 weeks while monitoring for possible side effects, but stop if there is no response to treatment after 12 weeks [17]. It inferred that isoflavones were generally safe and could have beneficial effects in some people. Taku et al. also felt that isoflavones were a reasonable alternative for women suffering from hot flashes but could not, or would not, take HRT [61].

We have yet to fully understand the biological pathways of soy, isoflavones, their metabolites, and how they interact with the human body. An in vitro study showed that isoflavones act as estrogen antagonists before menopause due to the high levels of endogenous estrogens circulating in the body, and act as estrogen agonists after menopause due to the low-estrogen environment [3]. However, there are still many unanswered questions. For example, we still do not know which active component of isoflavones provide the estrogen-like benefits, and studies have reflected this fact by experimenting on various isoflavone extracts or different parts of the soybean [62]. Studies that used whole soy foods generally estimated their total isoflavone intake to be greater (3.0–3.5 mg per gram of soy) than studies that used purified soy proteins (1.0–1.5 mg/g) [20]. On the other hand, it is also possible that substances in soy apart from isoflavones exert beneficial effects as well. In studies where isoflavones have been removed from soy products compared to soy extracts containing isoflavones, the results were similar [63]. Even more confusing is a systematic review that showed synthetic isoflavones to be superior to soy isoflavones in reducing hot flashes [27]. Overall, the evidence seems to suggest that soy, isoflavones, and their metabolites are mediated by both estrogen-dependent and estrogen-independent pathways [17].

Conflicting results are also seen when trying to standardize the isoflavone levels in Asian and other women. Reed et al. concluded that hot flashes were least common in Asian people regardless of soy intake, but did point out they were unable to account for equol-producing status in their study [10]. Burton and Wells found that neonatal exposure to phytoestrogens in rats had uterotrophic effects [53], and many other human studies have identified early life exposure as a prerequisite for dietary isoflavone benefit, postulating an epigenetic alteration on gene expression [17]. 

Due to this uncertainty of isoflavone action, the optimal dosage of isoflavones has also not been standardized across studies. Pharmacokinetic studies revealed that S-equol exposure was linear with dose, and was better administered at twice-daily doses [18]. As previously established, the total amount of isoflavones required for symptom relief in humans, expressed in aglycone equivalent weight, was approximately 40–50 mg/day [64]. No study has suggested an upper limit. Utian et al. found twice-daily rather than once-daily doses of S-equol to be more effective [65]. Crawford et al. found twice-daily dosing of isoflavones provided greater relief of hot flash than once-daily, but more frequent dosing may provide benefit for equol producers only [66]. In contrast to these studies, however, is glabridin, another isoflavone which has a biphasic rather than linear effect on human endometrial adenocarcinoma (Ishikawa) cells [67]. It therefore seems that S-equol and different isoflavone compounds are unique in their effects, and need to be discussed separately.

## 5. Conclusions

In the literature we reviewed, isoflavones reduce hot flashes even accounting for placebo effect, attenuate lumbar spine BMD loss, may show beneficial effects on systolic blood pressure during early menopause, and may improve glycemic control in vitro. On the other hand, the effect of isoflavones on GPER positive cells should be further explored. There are currently no conclusive benefits of isoflavones on urogenital symptoms and cognition.

Although isoflavones will never be as effective as hormone therapy in relieving menopausal symptoms, a survey found that 70% of women would be “satisfied with a nonhormonal intervention that provided at least a 50% reduction in hot flashes” [61]. The safety profile of isoflavones combined with their benefit to overall health makes them a compelling treatment option for postmenopausal women unwilling or unable to take hormone replacement therapy.

In order to minimize study heterogeneity in future research, we urge standardization of the many variables involved in isoflavone trials, such as the time to menopause that may have crucial effect on the isoflavone response. Isoflavone aglycone content should be consistent across trials for better communication, prediction of therapeutic activity, and ensure reproducibility. In addition, outcome measures and study duration should be standardized, along with the metabolic profiles of participants. Finally, bigger sample sizes are needed to increase the validity and generalizability of results.

## Figures and Tables

**Table 1:** A brief summary of current evidence of studies regarding the effects of isoflavones. Bone mineral density (BMD), hormone replacement therapy (HRT).

Studies (Ref. No.)	Study Design	Contents	Main Results
**Hot flashes**			
[23] St Germain	RCT	soy	no difference
[24] Tice	RCT	isoflavone tablets	no difference
[25] Cancellieri	RCT	isoflavone from herbal supplement	isoflavones more effective than placebo
[26] Cheng	prospective study	isoflavones extracted from soya bean	isoflavones more effective than placebo
[9] Welty	RCT, crossover	soy nut	soy more effective than placebo
[27] Thomas	systematic review	natural vs. synthetic isoflavones	synthetic or combination isoflavones more effective than natural soy
[28] Washburn	randomized crossover trial	soy protein	soy protein more effective than placebo
[29] Khaodhiar	RCT	daidzein-rich isoflavone aglycones	daidzein-rich isoflavone aglycones more effective than placebo
[30] Cianci	observational prospective study	calcium, vitamin D3, inulin, soy isoflavones	soy supplement + inulin effective
[31] Carmignani	RCT	soy vs. HRT	HRT more effective than soy; both are superior to placebo
[32] Bolanos-Dıaz	meta-analysis	soy extracts vs. HRT	HRT more effective than soy extracts; both are superior to placebo
[33] Amato	multicenter RCT	aglycone hypocotyl soy isoflavone	no difference
[34] Daily	systematic review, meta-analysis	soy isoflavone and equol	equol or isoflavone in equol-producers more effective than placebo
[19] Newton	observational study	equol-producer status	soy in equol-producers more effective than non-producers
[12] Lambert	RCT	red clover extracts	red clover extracts more effective than placebo
**BMD**			
[36] Ma	meta-analysis	isoflavone	increase spinal BMD
[33] Amato	multicenter RCT	aglycone hypocotyl soy isoflavone	slow BMD loss
[35] Lambert	systematic review and meta-analysis	isoflavone aglycone	preserve BMD
**CV**			
[37] van der Schouw	prospective study	food phytoestrogens	low dose phytoestrogen not protective
[38] Nestel	randomized crossover trial	purified soybean extract	may improve systemic arterial compliance
[26] Cheng	prospective study	isoflavones extracted from soya bean	no difference in lipoprotein lipids
[40] Wong	RCT	soy hypocotyl isoflavones	no effect on nitric oxide metabolism or blood pressure
[41] Suparto	animal study	soy protein	HRT + soy harmful, soy or HRT not beneficial
[42] Sathyapalan	double blind randomised study	soy protein +/− soy isoflavone	soy protein with isoflavones improved CVR markers compared to soy protein alone
**Metabolic syndrome**			
[43] Stuenkel	randomized clinical trial	isoflavone supplements	loss of weight and fat mass, but interpretation difficult
[44] Mueller	in vitro study	PPARgamma binding and transactivational activity	red clover extracts may be used to treat metabolic syndrome
**Cancer risk**			
[46] Hirose	case-control study	soy products as part of daily intake	lower risk of breast cancer in premenopausal women
[47] Alipour	case-control study	soy extracts	soy extracts may cause benign changes in breast
[49] Kang	cohort study	dietary intake of soy isoflavones	lower recurrence in postmenopausal women with estrogen- and progesterone-receptor positive breast cancers receiving anastrazole therapy after surgery
[50] Shin	case-control study	dietary soyfood and isoflavone intake	reduced risk for overall colorectal cancer
**Urogenital tract**			
[52] Reed	RCT	black cohosh or dietary soy	no effect on vaginal cytology
[51] Waetjen	prospective cohort study	dietary intake of isoflavones	no effect on stress or urge incontinence
[54] Vitale	prospective, randomized, placebo-controlled study	isoflavones, calcium, vitamin D, inulin	improves sexual function
**Cognition**			
[55] Greendale	cohort study	dietary phytoestrogens	better processing speed, but worse verbal memory
[56] Clement	systematic review	isoflavones and soy	may improve cognition
